# Dual Role of Indoles Derived From Intestinal Microbiota on Human Health

**DOI:** 10.3389/fimmu.2022.903526

**Published:** 2022-06-17

**Authors:** Xuewei Ye, Haiyi Li, Komal Anjum, Xinye Zhong, Shuping Miao, Guowan Zheng, Wei Liu, Lanjuan Li

**Affiliations:** ^1^ State Key Laboratory for Diagnosis and Treatment of Infectious Disease, Collaborative Innovation Center for Diagnosis and Treatment of Infectious Diseases, Zhejiang University, Hangzhou, China; ^2^ Department of Basic Medical Sciences, Shulan International Medical College, Zhejiang Shuren University, Hangzhou, China; ^3^ Department of Medicine and pharmacy, Ocean University of China, Qingdao, China; ^4^ Key Laboratory of Endocrine Gland Diseases of Zhejiang Province, Hangzhou, China; ^5^ Otolaryngology & Head and Neck Center, Cancer Center, Department of Head and Neck Surgery, Zhejiang Provincial People’s Hospital (Affiliated People’s Hospital, Hangzhou Medical College), Hangzhou, China; ^6^ Institute of Plant Protection and Microbiology, Zhejiang Academy of Agricultural Sciences, Hangzhou, China

**Keywords:** indole, inflammation, intestinal flora, indoxyl sulfate, dual role

## Abstract

Endogenous indole and its derivatives (indoles), considered as promising N-substituted heterocyclic compounds, are tryptophan metabolites derived from intestinal microbiota and exhibit a range of biological activities. Recent studies indicate that indoles contribute to maintaining the biological barrier of the human intestine, which exert the anti-inflammatory activities mainly through activating AhR and PXR receptors to affect the immune system’s function, significantly improving intestinal health (inflammatory bowel disease, hemorrhagic colitis, colorectal cancer) and further promote human health (diabetes mellitus, central system inflammation, and vascular regulation). However, the revealed toxic influences cannot be ignored. Indoxyl sulfate, an indole derivative, performs nephrotoxicity and cardiovascular toxicity. We addressed the interaction between indoles and intestinal microbiota and the indoles’ effects on human health as double-edged swords. This review provides scientific bases for the correlation of indoles with diseases moreover highlights several directions for subsequent indoles-related studies.

## 1 Introduction

A person’s intestine is filled with trillions of bacteria, far exceeding any other microbial population on the body’s surface (1–3). As a complex microecosystem, intestinal bacteria are closely related to the host and play a core role in regulating physiological functions related to nutrition, immune system activation, and host defense (4). One of the main ways the intestinal flora interacts with the host is through metabolites (5, 6). Short-chain fatty acids (7–9) and bile acids (10) are metabolites from intestinal flora that contribute to intestinal health. Recent data indicate that indoles generated by tryptophan (Trp) metabolism are essential factors in intestinal homeostasis, closely related to intestinal microecology and human health (11, 12).

**Figure 1:**
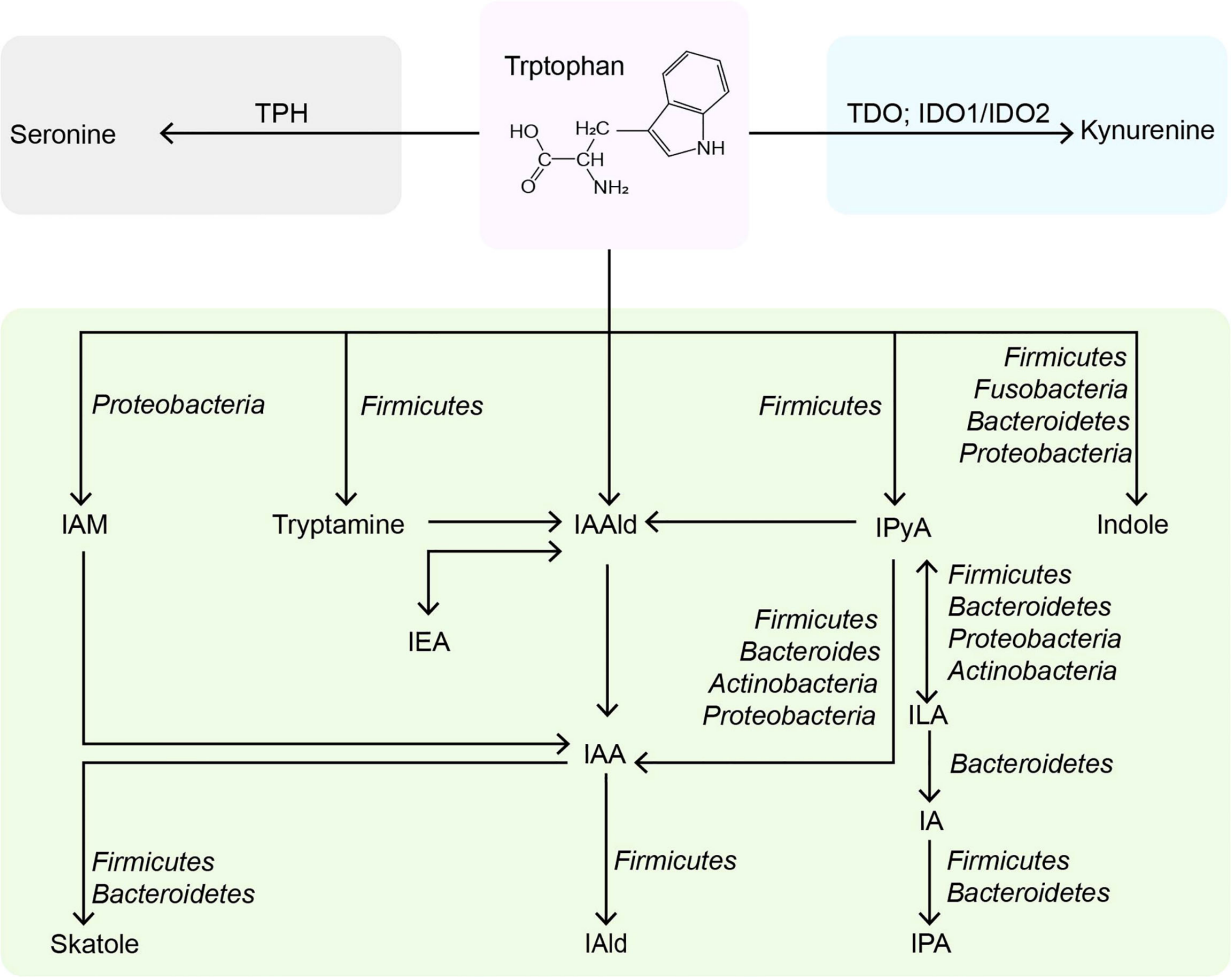
Metabolic pathways of tryptophan in the intestine. TPH, tryptophan hydroxylase; TDO, tryptophan 2,3-dioxygenase; IDO, indoleamine 2,3-dioxygenases; IAM, indole-3-acetamine; IAAld, indole-3-acetaldehyde; IPyA, indole-3-pyruvate; IEA, indole-3-ethanol; IAA, indole-3-acetate; ILA, indole-3-lactate; IAld, indole-3-aldehyde; IA, indole-3-acrylate; IPA, indole-3-propionate.

Trp is an essential nutrient mainly consumed through external foods but cannot be synthesized endogenously. Trp plays an essential part in regulating intestinal immune tolerance, maintaining symbiotic microbial homeostasis, and suppressing inflammation (13). Dietary tryptophan deficiency led to impaired immune function and altered gut in mice (14). Tryptophan-containing diets reduced inflammatory response and improved colonic inflammation and severity induced by sodium dextran sulfate in mice (15). The role of dietary Trp in modulating host immunity was closely related to the endogenous metabolites of Trp (16). Trp metabolism is a multi-path and complex process occurring in the host and its intestinal symbiotic microbiota, which has been extensively studied. In addition to being used for protein synthesis, Trp in the gut is metabolized through three main pathways: 1) the kynurenine pathway (95% of ingested Trp). 2) bacterial Trp metabolism (4-6%). 3) the serotonin pathway (1-2%) (**Figure 1**). Most of the existing reviews focus on Trp (17–19) and kynurenine (20–22), and few reviews systematically describe the properties of indoles (23).

Endogenous indoles in the human body are mainly the bacterial catabolite of Trp. Bacterial-derived indoles are valued signal molecules between bacterial cells and play a prominent role in the microbial community that influences spore formation, plasmid stability, drug resistance, biofilm formation, and toxicity (24–29). Additionally, indoles strengthen the efficacy of intestinal epithelial cells (IECs) and suppress inflammation, regulate gut insulin secretion, and maintain the youth and health in animals, which may make it useful for humans (30–32). Indole skeleton is considered one of the most promising heterocyclic compound types with significant physiological and biological activities, including anti-cancer, anti-convulsant, anti-microbial, anti-tuberculosis, anti-malaria, and anti-viral (33, 34). Compared with indole from enterobacteria, synthetic indole compounds are more widely used in medicine, pesticides, food additives, etc., but they have toxicity and side effects (35, 36). Indole synthesized by enterobacteria has extensive benefits to human health and plays an increasingly important role in the intestinal tract (11). Thus, we reviewed the latest findings related to indoles from intestinal microbiota, focusing on how they promote intestinal immune homeostasis. We also summarized the beneficial effects of indoles on intestinal inflammation and other systemic diseases, and put forward the potentially toxic side effects. The purpose is to understand and reveal the further influence of indoles on human health. Besides, it could provide a reference for treating related diseases and developing new drugs in the future.

## 2 Indoles Derived From the Intestinal Flora

Trp is the primary precursor of indole derived from the intestinal flora. Most of the Trp is absorbed in the small intestine, and about 4% ~ 6% of Trp is catabolized into indoles by intestinal flora in the colon (37, 38). Typically, Gram-negative and Gram-positive bacteria, such as *Escherichia coli E. coli*), *Clostridium* spp., and *Bacteroides* spp., expressing tryptophanase, catalyze the direct conversion of Trp to indole (39). The widely studied *E. coli* import Trp through TnaB, and Trp is reversibly degraded into indole, pyruvic acid, and ammonia by tryptophanase TnaA (40). It has been found that more than 85 kinds of bacteria (Gram-negative and Gram-positive) metabolize dietary Trp to indole by tryptophanase (41). Indole affects bacterial physiology in a concentration-dependent manner (42). For instance, 0.5 mM affects bacterial movement (43), biofilm formation (44), and persister cell formation (45); 1-2 mM indole concentration influences the expression of multidrug exporters (46) and the secretion of specific virulence factors (47); 3-5 mM will inhibit cell division (48) and affect plasmid stability (49). And the concentration of indole is limited by the amount of exogenous Trp, so the concentration of indole in human feces is relatively high, ranging from 0.25 mM to 1.1 mM (31). The Kovács method is the most commonly used to detect the indole concentration in various biological samples (50). Still, it is complicated to operate and lacks specificity, so it is necessary to develop a simpler and more efficient quantitative method in the future. Although TnaA can metabolize the most Trp to indole, its reaction is only a small part of the microbial degradation pathway of Trp. Trp can also be hydrolyzed by decarboxylases, such as *Ruminococcus gnavus* and *Clostridium sporogenes* (*C. sporogene*), which produce the β-arylamine neurotransmitter tryptamine by decarboxylating Trp through the action of tryptophan decarboxylase (51). Tryptamine can regulate intestinal motility and immune function (52). It can also induce intestinal endocrine cells to release serotonin (53), which has impacts on human emotions, immunity, and bone development, and also involves the pathology of many diseases, including inflammatory bowel disease (IBD) and cardiovascular diseases (54).

Additionally, *C. sporogenes* can transform Trp into idole-3-pyruvic acid (IPyA), which is catalyzed by an indole-3-pyruvate decarboxylase to produce indole-3-acetaldehyde (IAAld) (11). Then, IAAld is decarboxylated by members of the phylum *firmicutes*, *proteobacteria*, *bacteroides*, and *actinomycetes* to produce indoleacetic acid (IAA) (55, 56). IAA is the precursor of indole-3-aldehyde (IAld) and 3-methylindole (skatole). The skatole is formed by decarboxylation of IAA, and *Bacteroides thetaiotaomicron*, *Eubacterium rectale*, and *Butyrivibrio fibrisolvens* have been proved to produce it. IAld is produced by the catabolism of *Lactobacillus acidophilus* (*L. acidophilus*), *L. murinus*, *L. reuteri*, and *L. johnsonii* in the firmicutes (57–59). Moreover, IPyA can produce indole-3-lactic acid (ILA), indoleacrylic acid (IA), and indole 3-propionic acid (IPA) through reduction and dehydration. Intestinal bacteria containing phenyllactate dehydrogenase (fldH) convert IPyA into ILA through a reduction reaction. ILA can produce IA through dehydration of *Peptostreptococcus russellii* (*P. russellii*), *P. anaerobius* and *P. stomatis*, phenyllactic dehydratase (fldBC) and its activator *fldI* are involved in this process (11). The acyl-CoA dehydrogenase enzyme converts IA to IPA, the final product of Trp reduction metabolism. Other bacteria, such as *Lecheveria aerocoloniae*, can make IPA by deaminating Trp with an amino acid oxidase (60). Among the enzymes listed above, *fldAIBC* should be worth mentioning. This gene cluster is only found in *P. russellii* and is preserved across the *Peptostreptococcus* genus (61). Although *P. anaerobius* also encodes the complete *fldAIBC* gene cluster, the concentrations of IPA and IA produced by it are significantly reduced. Furthermore, the *fldAIBC* cluster in *C. sporogenes* is identical to this gene cluster, which is assumed to be responsible for the conversion of Trp into ILA and IPA in *C. sporogenes* (39, 62). Additionally, comparable gene clusters have been discovered in *C. cadaveris*, *C. botulinum*, and *P. anaerobius*, indicating that they can produce IPA (62). At the same time, the presence of FLDC, a homologous cluster of the *fldBC* gene cluster, was discovered to be a reliable marker of IPA-producing bacteria (62). We show the metabolic pathway of indoles in the intestine and related bacteria (detailed to the phylum) in **Figure 1**.

Trp metabolism is complex, and numerous bacteria strains are involved in the manufacturing of indole derivatives (56). However, there are still many undiscovered strains that can catalyze Trp. To identify microorganisms and microbial genes involved in Trp metabolism regulation, it seems a feasible strategy to combine metabolomics with metagenomics and/or metatranscriptomics. Many metabolites mentioned above have been proved to be beneficial to the host, and they have important effects on the intestinal barrier function and even human health (61, 63).

## 3 Influence on Intestinal Function

**Figure 2:**
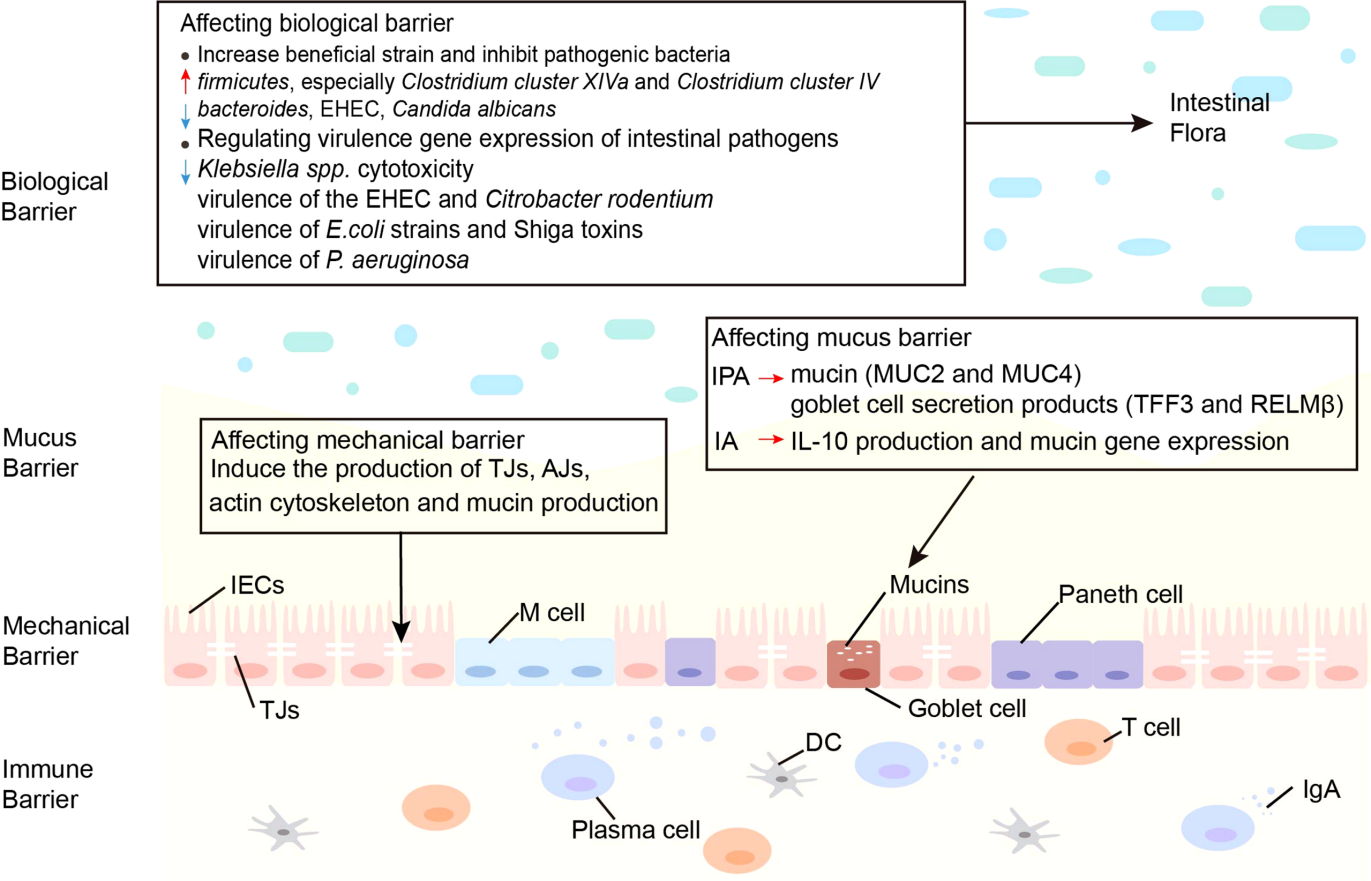
Effects of indoles on three barriers. Indoles affect the biological barrier by increasing beneficial bacteria, inhibiting pathogenic bacteria and regulating virulence gene expression of intestinal pathogens. Indoles enhance intestinal epithelial cell function by regulating several genes involved in mechanical barrier formation. Indoles increase mucin and goblet cell secretion products, which strengthen the mucus barrier. AJs, adherens junctions; DC, dendritic cell; *E. coli*, *Escherichia coli*; EHEC, enterohemorrhagic *escherichia coli*; IA, indoleacrylic acid; IECs, intestinal epithelial cells; IgA, immunoglobulin A; IL-10, interleukin-10; IPA, indole 3-propionic acid; MUC, mucin; M cell, membranous cell; *P. aeruginosa*, *Pseudomonas aeruginosa*; TJs, Tight junctions.

The function of the intestinal barrier is strongly linked to intestinal health and plays a critical role in animal health. The gut barrier isolates the host from the microorganisms in the intestinal cavity and limits the movement of microorganisms and molecules from the intestinal lumen (64). The intestinal mucosal barrier consists of 4 main components: immune barrier, biological barrier, mechanical barrier, and mucus barrier (65). In the intestine, lymphoid tissue and immune cells make up the immunological barrier. The biological barrier is made of intestinal flora. The mechanical barrier consists of tight junctions (TJs) and the underlying gut epithelium. Chemicals such as lysozyme and digestive enzymes secreted by the intestine form the mucus barrier (**Figure 2**). An intact mucosal barrier system prevents microorganisms and products from migrating into the blood (66). Once the gut barrier is damaged and intestinal permeability increases, bacteria and their products, such as endotoxins, will translocate and activate the mononuclear macrophage system, promoting the production of a large number of inflammatory elements, such as interleukin (IL)-6 and tumor necrosis factor-α (TNF-α) leads to a chronic microinflammatory state (67, 68). Indoles are crucial in controlling intestinal barrier efficacy, including modulation of inflammatory and immunological responses, reduction of epithelial permeability, mucus production, and TJ formation. The principal effects of indoles on the intestinal immune barrier are summarized in **Table 1**.

**Table 1:** Effects of indoles on intestinal immune barrier.

Type	Pathways that affect the immune barrier	Related cytokines/cells	Reference
Indole	Decreases IL-8 secretion, inhibits NF-κB activation, increases IL-10 secretion	IL-8, NF-κB, IL-10	(31)
IPyA	Promotes Tr1 differentiation, reduces Th1 cells, changes the composition of the MLN DC subgroup	Tr1, Th1, CD103^-^CD11b^+^ DCs, CD103^+^CD11b^-^DCs	(69)
IA	Increases IL-10 secretion, inhibits TNF production; activates NRF2-ARE pathway, inhibits IL-6 and IL-1β secretion	IL-10, TNF, IL-6, IL-1β	(61)
IAA	Increases CD103^+^/CD11c^+^ abundance and IL-22 expression	CD103^+^/CD11c^+^, IL-22	(70)
	Promotes IL-35^+^ B cells production	IL-35^+^ B	(71)
	Activates IL-22/STAT3 signaling pathway, increases antimicrobial peptides production	IL-22	(72)
ILA	Activates AhR in CD4^+^ T cells, decreases ThpoK, allows CD4^+^ T cells to differentiate into CD4^+^CD8αα^+^ DPIELs	CD4^+^CD8αα^+^ DPIELs	(59)
	Decreases IL-8 expression, activates AhR target gene and NRF2 target gene	IL-8	(73)
IPA	Inhibits TNF-α production	TNF-α	(74)
	Increases IL-10R1 production	IL-10R1	(75)
IAld	Increases IL-22 expression	IL-22	(58)
	Activates IL-22/STAT3 signaling pathway, inhibits IL-1β and TNF-α secretion	IL-22, IL-1β, TNF-α	(76)

IL, interleukin; NF-κB, nuclear factor κB; IPyA, idole-3-pyruvic acid; Tr1, t regulatory cell 1; Th1 cells, t helper cell 1; MLN, mesenteric lymph nodes; DC, dendritic cell; IA, indoleacrylic acid; TNF, tumor necrosis factor; NRF2, erythroid 2-related factor 2; ARE, antioxidant response element; IAA, indoleacetic acid; STAT, signal transducer and activator of transcription; ILA, indole-3-lactic acid; AhR, aryl hydrocarbon receptor; DPIELs, double-positive intraepithelial T lymphocytes; IPA, indole 3-propionic acid; IAld, indole-3-aldehyde.

### 3.1 Affecting the Intestinal Immune Barrier

#### 3.1.1 Indole Enhances Immune Barrier

The use of indole as a therapy for non-steroidal anti-inflammatory drugs (NSAIDs) enteropathy was investigated (77). Indole decreases the fecal calprotectin concentrations and the infiltration of neutrophils in the spleen and mesenteric lymph nodes induced by indomethacin. Combining indole with indomethacin leads to a reduction in NSAID-induced mucosal transcriptome changes. Fecal calprotectin is a neutrophil-specific biomarker of intestinal inflammation. And neutrophils are considered the key factor in the pathogenesis of NSAIDs enteropathy, which can cause inflammation and tissue damage by releasing a variety of inflammatory mediators (78). This indicates that indole can reduce the intestinal inflammation caused by indomethacin in mice, and has a potential immunomodulatory effect on NSAID enteropathy.

Bansal et al. found that indole reduced the creation of the pro-inflammatory cytokine IL-8 and the expression of nuclear factor κB (NF-κB) activated by chemokine TNF-α and induced the secretion of the anti-inflammatory cytokine IL-10 (31). Studies have shown that symbiotic bacteria can limit the activation of NF-κB induced by *Salmonella typhimurium* and reduce inflammation in mice (79). Therefore, indole may be a signal that probiotics reduce intestinal inflammation.

#### 3.1.2 Indole Derivatives Enhance Immune Barrier

IPyA is an aromatic pyruvic acid produced by the action of aromatic amino acid transaminases and is a precursor of ILA, IAA, and IAAld. IPyA had a substantial anti-inflammatory impact on the colon, in the IBD mouse model. IPA administration modified the constitution of T-cell subsets in the colonic lamina propria lymphocytes (LPL) and dendritic cells (DCs) subsets in the mesenteric lymph nodes (MLN). In T cell-mediated colitis model, IPyA induced T regulatory cell 1 (Tr1) differentiation but not Foxp3^+^ Treg differentiation in the colonic LPL (69). Tr1 secretes a large amount of IL-10 and is important for intestinal immunological homeostasis (80). Simultaneously, IPyA reduces the frequency of Th1 cells by attenuating the ability of MLN DC to induce Th1 cell differentiation. Th1 cells are involved in the etiology of colitis (81), it appears that IPA decreases colonic inflammation by inhibiting Th1 cell production. IPyA administration modified the constitution of MLN DC subpopulations, mainly by decreasing the frequency of CD103^-^CD11b^+^ DCs and increasing the frequency of CD103^+^ CD11b^-^ DCs in MLN. CD103^-^CD11b^+^DCs promote the differentiation of effector T cells that produce pro-inflammatory cytokines IFN-γ and IL-17. Besides, CD103^-^ DCs are greatly inflammatory during chronic colitis (82). This is sufficient to demonstrate that changing the composition of MLN DC subsets has an effect on the improvement of intestinal inflammation. IPyA suppresses colonic inflammation by boosting IL-10-producing T cells and lowering Th1 cells in the lamina propria of the colon. IPyA administration alters the composition of the MLN DC subpopulation, suggesting that IPyA has an important effect on improving the intestinal immune barrier.

IA of bacterial origin increased IL-10 production and reduced TNF production in an LPS-stimulated co-culture (a co-culture system consisting of mini-gut spheroid cultures and BMDMs) (61). IL-10 is crucial for cupped cells to keep producing mucin (MUC) (83). According to research, IA may have a considerable anti-inflammatory effect on the colon. In addition to the activation of aryl hydrogen receptor (AhR), treating human PBMCs with IA activated nuclear factor erythroid 2-related factor 2 (NRF2)- antioxidant response element (ARE) pathway and secreted less IL-6 and IL-1β. NRF2 is a transcription factor that stimulates the ARE pathway in cells, inhibits the pro-inflammatory signaling pathway, and activates the AhR signaling pathway (84, 85). Furthermore, RNA sequencing demonstrated that IA therapy resulted in the activation and/or differentiation of innate immune cells (e.g., CD14, CCL2, MT2A, CYBB, IL6, and PTAFR), as well as genes involved in inflammation and oxidative stress (e.g., FPR2, LRRC25, CPM, MS4A7, and SLC7A7) (61). The findings above point to IA inducing potent anti-inflammatory and antioxidant effects in humans.

Production of IAA by *Bacteroides ovatus* increases the abundance of CD103^+^/CD11c^+^ immune populations (70), and immunological cells that are CD103^+^/CD11c^+^ are critical for maintaining intestinal immune homeostasis and inducing tolerogenic immune responses (86). In addition, IAA binds to the AhR on DCs and drives the production of IL-22. In individuals with IBD, IL-22 stimulates epithelial regeneration and decreases inflammation, and it plays a key role in the regulation of intestinal inflammation (87). *In vivo*, activation of the IL-22 receptor increases the expression of genes involved in immune surveillance, epithelial barrier function, inflammation, and homeostasis (88). Indole production by other *Bacteroides* besides *Bacteroides ovatus* can also activate AhRs on immune cells, leading to IL-22 production. Still, no research on *Bacteroides*, indole synthesis, and IL-22 secretion has been conducted, so further studies are needed to verify the possibility that IAA produced by other *Bacteroides* activates IL-22 and reduces colitis. In the presence of LPS, Reg4 expressed in intestinal epithelial cells maintains immune homeostasis by increasing the proportion of *Lactobacillus* and its metabolite IAA, which promotes the production and accumulation of IL-35 ^+^ B regulatory (Breg) cells in intestinal tissues (71). IL-35-producing Breg cells are key immune regulators of many diseases, including autoimmune and infectious diseases and cancer progression (89). IL-35 expression is dysregulated in inflammatory autoimmune diseases such as IBD, multiple sclerosis, type 1 diabetes, and autoimmune hepatitis (90). These results suggested that IAA can regulate the differentiation and production of IL-35^+^ cells, and affect the intestinal immune barrier. After being treated with Trp (LAB + Trp), *Lactobacillus plantarum* KLDS 1.0386 elevated IAA levels in the colon. IAA further upregulated the expression of AhR mRNA to stimulate the IL-22/signal transducer and transcription (STAT) 3 signaling pathwayactivator, increased the formation of antimicrobial peptides like regenerated islet-derived protein 3β and regenerated islet-derived protein 3γ, and improved gut immune function (72).

*Lactobacillus reuteri* produces ILA, an indole derivative of Trp, which activates AhR in CD4^+^ T cells and downregulates the transcription factor ThpoK, allowing CD4^+^ T cells to differentiate into CD4^+^CD8αα^+^ double-positive intraepithelial T lymphocytes (DPIELs) with immunomodulatory functions (59). Through these mechanisms, ILA promotes intestinal barrier function and reduces inflammation. *Bifidobacterium longum subsp. Infantis* living in the gastrointestinal tract of breast-fed infants can also produce ILA, which significantly attenuates TNF-α and LPS-induced increases in the proinflammatory cytokine IL-8 in intestinal epithelial cells. ILA also increases mRNA expression of the AhR-target gene CYP1A1 and NRF2-targeted genes glutathione reductase 2, superoxide dismutase 2, and NAD(P) H dehydrogenase, which may be important modulators of intestinal inflammation in breastfeeding infants (73). In addition, another study showed that ILA secreted by *B. infantis* has anti-inflammatory impacts on the immature intestine (91). These data provide important insights for the production of ILA-producing probiotics and dietary recommendations.

IPA downregulated the intestinal epithelial cell-mediated inflammatory cytokine TNF-α, while upregulating the ligand protein-encoding mRNAs (74), thereby regulating the intestinal barrier function and relieving intestinal inflammation. IPA also regulated intestinal immune homeostasis by significantly inducing IL-10 receptor ligand-binding subunit (IL-10R1) on intestinal epithelial cells through activation of AhR (75). IL-10R1 is a receptor for the anti-inflammatory cytokine IL-10, and IL-10 sends anti-inflammatory signals through IL-10R1 that inhibits the excessive release of pro-inflammatory mediators from various cells, including IEC. This further illustrates the protective effect of IPA on the intestinal immune barrier.

IAld produced by *Lactobacillus* contributed to AhR-dependent IL22 transcription (58), and the IL-22 producer inhibited inflammation and protected the immune physiology of the mucosal surface. IAld could replace probiotics to protect and maintain mucosal integrity during infection or chemical injury. IAld might be used as a supportive therapy during flora processing and intestinal flora dysbiosis. *Lactobacillus* also stimulated IL-22 secretion by LPL through IAld-induced AhR, which activated the pSTAT3 pathway and inhibited IL-1β and TNF-α secretion to protect the mucosal immune barrier (76).

### 3.2 Affecting the Intestinal Biological Barrier

#### 3.2.1 Increase Beneficial Strains and Inhibit Pathogenic Bacteria

The gut microbiome is dominated by gram-negative bacteria when taking NSAIDs, a shift that can cause intestinal damage. Co-administration of indole and indomethacin can maintain or even increase the important members of *firmicutes*, especially *C. cluster XIVa* and *C. cluster IV*, which are crucial to intestinal homeostasis. It seems that it can prevent any increase of *bacteroides*. Intestinal mucosal injury can be reduced with this change (77). Indole may increase the resilience of HCT-8 cells to norepinephrine-mediated *enterohemorrhagic Escherichia coli* (EHEC) settlement through multiplying expression of the MUC gene (31). IAld activates AhR to produce IL-22, and IL-22 regulated mucosal responses that allow mixed microbial communities to survive and suppress *Candida albicans* colonization (58).

#### 3.2.2 Regulating Virulence Gene Expression of Intestinal Pathogens

Indoles modulate virulence factors in a variety of intestinal bacteria. Indole enhances the conversion of tilivamycin to tilivalline. Both are produced by *Klebsiella* spp., with the difference that tilivalline is an indole analogue with reduced cytotoxicity, and tilivalline binds to upregulate progesterone X receptors (PXR) reactive detoxification genes and inhibits microtubule protein directed toxicity. Thus, indole alleviates *Klebsiella* spp. cytotoxicity in a multifunctional manner (92). Indole generated by the metabolism of intestinal probiotics has a high concentration in the intestinal lumen, which reduces the demonstration of the pathogenic gene of the intestinal germ EHEC and *Citrobacter rodentium* (29). Indole is also used as ToxR agonist to regulate virulence gene expression and biofilm production of *Vibrio cholerae* in the intestine (93). Indole, ICA, and IAA also down-regulate the virulence of pathogenic *E. coli* strains and the production of Shiga toxins (94). Moreover, the virulence gene expression of *Pseudomonas aeruginosa* is significantly altered by indole and 7‐hydroxyindole, which lower virulence factors and decrease swarming motility. *P. aeruginosa* less colonizes the guinea pig lung, and there is better clearance in the gastrointestinal tract after treatment with 7-hydroxyindole (95). As a result, indoles may be helpful as therapeutic agents against pathogens such as EHEC.

### 3.3 Affecting the Intestinal Mechanical Barrier

Indole enhances the barrier function of ECs *in vitro* by inducing the expression of many genes involved in IECs, including TJ, adherens junction (AJ), actin cytoskeleton, and MUC formation (96). The human intestinal epithelial cell line HCT-8 was exposed to indole to measure changes in its gene expression. Results showed that high doses of indole enhanced molecular profiles associated with MUC production and mucosal barrier strengthening and interepithelial resistance in polarized cells of the intestinal epithelium, HCT-8 (31). Thus, Indole strengthens the mechanical barrier.

### 3.4 Affecting the Intestinal Mucus Barrier

IPA enhances the mucus barrier function by increasing MUC2 and MUC4, as well as goblet cell secretory products (TFF3 and RELMβ) (97). IPA also promotes IL-10 release, and the signal transduction of IL-10 enhances the mucus barrier function and maintains the steady-state of epithelial cells (75). IA from bacteria enhances the production of IL-10 and MUC gene expression, and IL-10 is critical for maintaining MUC production in goblet cells. Therefore, IA indirectly affects the mucus barrier through IL-10 (61).

## 4 Mechanism of Indoles Regulating Intestinal Tract

**Figure 3:**
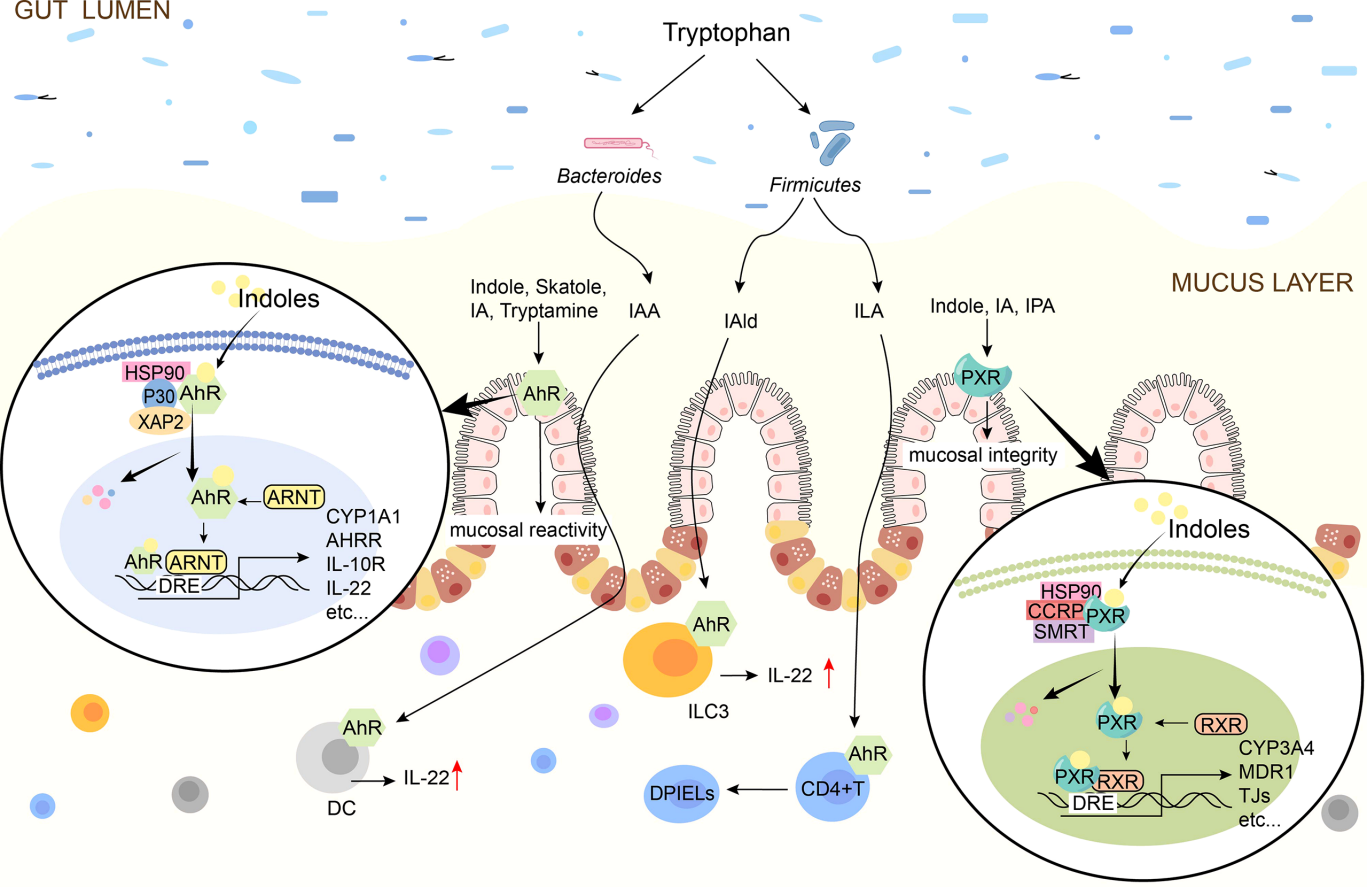
Schematic diagram of indoles signal pathway. Indole, Skatole, IA, Trytamine, IAA, IAld, ILA are AhR ligands. Indoles activates AhR to promote mucosal immunity, and AhR is present on intestinal epithelial cells and immune cells such as ILC3, DC. Indole, IA, IPA are the ligands of PXR and they activate PXR to affect tight junctions. In the absence of ligand, AhR/PXR exists in cytoplasm as an inactive complex. After indoles binding, AhR/PXR enters the nucleus, forms a dimer with ARNT/RXR, and then induces the expression of immune-related genes. IAA, indole-3-acetate; ILA, indole-3-lactate; IAld, indole-3-aldehyde; IA, indole-3-acrylate; IPA, indole-3-propionate; AhR, aryl hydrocarbon receptor; PXR, pregnane X receptor; TJ, tight junctions; IL, interleukin; DC, dendritic cells; DPIELs, double-positive intraepithelial T lymphocytes; ILC3, Group 3 innate lymphoid cells; HSP90, heat shock protein 90; P23, HSP90 co-chaperone p23; XAP2, X-associated protein 2; ARNT, AhR nuclear translocator.

Indoles are critical molecules in host-microorganism interaction, and most of them play a protective role in the intestinal barrier. It mainly regulates the mucosal immune response by activating AhR and regulates the mucosal integrity by activating PXR, thus regulating intestinal homeostasis (98, 99). The mechanism by which indole regulates the intestinal barrier *via* AhR or PXR is shown in **Figure 3**.

### 4.1 Intermediation of Aromatic Hydrocarbon Receptor

Indole, tryptamine, Skatole, IAA, IA, and other indolic acid derivatives are considered as ligands of AhR (100) and play immune roles by activating AhR (38, 101). AhR is a key member of the basic helical-helix superfamily and is found in immune cells, ECs, and endothelial cells of intestinal barrier tissues (56, 102). Inactive AhR exists in the cytoplasm and binds to 2 molecules of heat shock protein 90 (Hsp90), 1 molecule of X-associated protein 2 (XAP2), and 1 molecule of Hsp90 co-chaperone p23 (P23) to form complexes, once activated by ligands, the conformation of the complex changes (99). AhR enters the nucleus and joins with AhR Nuclear Translocator (ARNT) to produce a functional heterodimer transcription factor complex (98). The dioxin reaction element is a constant DNA sequence that the AhR/ARNT heterodimer binds to (DRE) (103). The recruitment of coactivators to AhR’s trans-activation domain occurs when AhR/ARNT binds to DRE, which initiates the indication of a series of downstream target genes, such as inflammatory and immune-related cytokines IL-22, IL-17and IL-10, thereby exerting immunomodulatory effects (104, 105).

The cytokine IL-10 is produced by AhR indole and ICA, promoting intestinal regeneration and differentiation of goblet cells by altering intestinal homeostasis, protecting the epithelial barrier, and limiting inflammation associated with bacterial product transport, primarily during aging (32). Accordingly, indole may be useful for treating age-related diseases including inflammation and epithelial barrier disruption. IL-10 signals are induced during inflammation through the IL-10R1, attenuating the proinflammatory mediators produced excessively in various cell types, including IECs, enhancing mucosal barrier function and leading to epithelial cell maintenance and homeostasis. The activity of IL-10R1 in the epithelium of an AhR is modulated by IPA and IAld (75), which has been linked to protective effects in colitis models and improves epithelial wound healing (**Table 2**).

**Table 2:** The mechanism of indoles regulating intestinal action through AhR.

Ligands	Indoles	The specific mechanism	Medium	Function	Reference
AhR	Indole	increase IL-10	IL-10	alter intestinal homeostasis by increasing intestinal cell renewal and promoting goblet cell differentiation	(32)
	IPA, IAld	modulate epithelial IL-10R1 activity in an AhR-dependent manner, IL-10 binding to IL-10R1, attenuate the overproduction of pro-inflammatory mediators intestinal epithelial cells		binds to IL-10R1 to enhance mucosal barrier function and maintain epithelial homeostasis. IL-10R1 activity is associated with protective effects in colitis models and promotes epithelial wound healing;	(75)
	IAld	promotes IL-22 *via* the AhR, activates innate lymphocytes	IL-22	anti-fungal colonization of *Candida albicans* and protection of mucous membranes from inflammation	(58)
	IAA	stimulates IL-22/STAT3 signaling pathway to activate Reg3γ gene expression		activates the immune system and strengthens the intestinal epithelial barrier to exert anti-inflammatory effects	(72)
PXR	IPA	down-regulating intestinal TNF-α and up-regulating connexin-coding mRNA	TLR4	improve the permeability and inflammation of intestinal barrier	(74)
	IAA	induce IL-35^+^ B cell production by activating PXR receptors and promoting IL35 release		maintain intestinal homeostasis	(71)

AhR, aryl hydrocarbon receptor; IL, interleukin; IAld, indole-3-aldehyde; IPA, indole 3-propionic acid; IAA, indoleacetic acid; STAT, transcription; Reg3γ, regenerated islet-derived protein 3γ; PXR, progesterone X receptors; TLR4, toll-like receptor 4; TNF-α, tumor necrosis factor-α.

IL-22 is an essential cytokine that protects the host from inflammatory damage. Its main function in the intestine is to maintain intestinal epithelial integrity and enhance defense mechanisms against bacterial pathogens (106). IAld stimulates the presentation of IL-22 in an AhR-dependent manner and activates innate lymphoid cells, which prevents fungal *Candida albicans* from colonizing the mucosa and inflaming it (58). IAA acts as an AhR ligand that promotes Reg3 gene expression *via* stimulating the IL-22/STAT3 signaling pathway, which activates the immune system and improves the intestinal epithelial barrier, resulting in anti-inflammatory benefits (72) (**Table 2**).

### 4.2 Modulation of Pregnancy-X Receptor

PXR is one of the members of the nuclear receptor superfamily, is widely expressed in intestinal tissues, can be activated by a multiplicity of endogenous and exogenous substances (107), and regulates intestinal mucosal integrity mediated by toll-like receptor 4 (TLR4) (108). Indoles activate PXR and induce anti-inflammatory responses (109). It has been found that IPA acts as a ligand for PXR *in vivo*, and IPA downregulates enterocyte TNF-α while upregulating ligand-encoding mRNA and increasing the manifestation of TJs that regulate intestinal barrier function in terms of intestinal permeability and inflammation (74). IAA combined with the PXR receptor can cause the production of IL-35^+^B cells, promoting potent anti-inflammatory cytokine IL-35, which maintains intestinal homeostasis (71). In addition, indole and indole-3-acetamide have also been suggested as PXR agonists (110) (**Table 2**).

## 5 Indoles Improve Diseases

**Figure 4:**
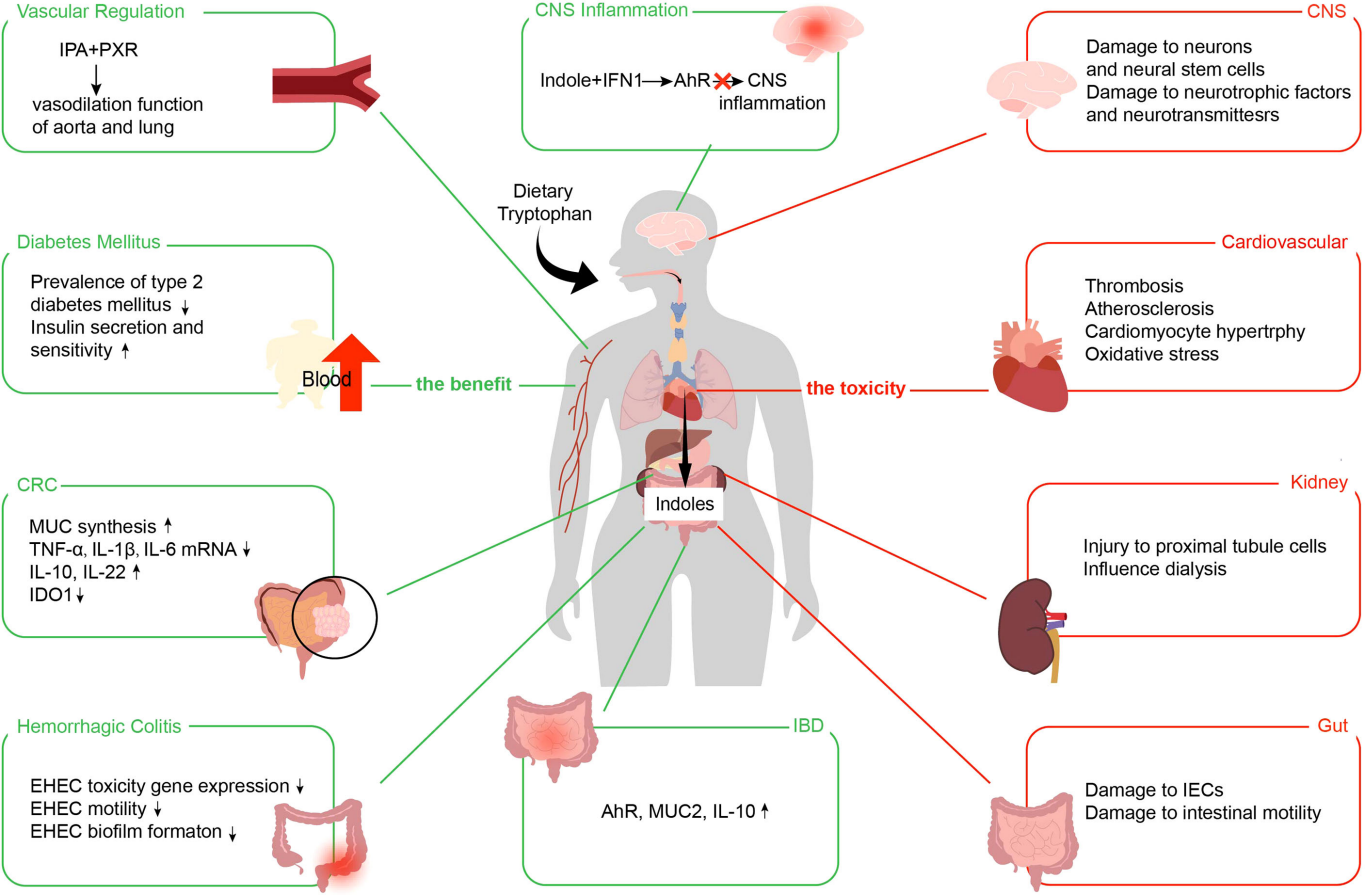
Benefits and toxic effects of indoles. The benefits of indoles use the green line. The toxicities of indoles use the red line. IPA, indole 3-propionic acid; PXR, pregnane X receptor; IFN, type I interferon; AhR, aryl hydrocarbon receptor; CNS, central nervous system; CRC, colorectal cancer; MUC, mucin; TNF-α, tumor necrosis factor-α; IL, interleukin; IDO1, indoleamine 2,3-dioxygenase; EHEC, enterohemorrhagic *Escherichia coli*; IBD, inflammatory bowel disease; IECs, intestinal epithelial cells.

Indoles are absorbed by intestinal epithelial cells and diffuse into the blood, thus circulating to the whole body and affecting various systems, such as the regulation of intestinal and related diseases. We summarize their effects on IBD, hemorrhagic colitis, colorectal cancer (CRC), diabetes mellitus, central system inflammation, and vascular regulation (**Figure 4**).

### 5.1 Indoles Relieves IBD

IBD is the general name of a group of diseases, including Crohn’s disease and ulcerative colitis, and it is an important chronic gastrointestinal inflammatory disease clinically (111). However, its pathogenesis is still unclear, and its treatment methods are limited. A large number of studies have shown that indoles play a certain role in relieving symptoms of IBD (101, 112).

IBD is linked to the mucus layer, and IBD patients have a thinner internal mucus layer and lower MUC2 glycosylation. It shows that IA treatment of murine-derived colonic spheroids significantly increases AhR activation and MUC2 gene expression. IA maintained its effect on MUC2 gene expression and increased IL-10 production even in the presence of LPS-stimulated co-culture. The generation of MUC by goblet cells is dependent on IL-10. As a result, IA produced from bacteria has the potential to increase IL-10 production and MUC gene expression, which may be advantageous to IBD patients (61).

The fecal samples from patients with IBD have been reported to induce less AhR activation than the fecal samples from healthy subjects, which has been ascribed to reduced amounts of IAA, a microbial AhR agonist, in the fecal samples from patients with IBD. Besides, research on mice lacking the IBD susceptibility gene Card9 showed that the absence of an altered microbiome capable of generating AhR agonists was linked to an increased risk of colitis (113). These data suggest that delivering adequate AhR agonists to the gut might be a promising technique for treating IBD. Indoles such as IAld have been proven to be effective AhR activators and may be effective immunomodulatory therapies for patients with IBD (58). Therefore, whether the products of indole metabolic pathway can be used as clinical drugs for IBD intervention is a scientific problem worth exploring.

### 5.2 Indoles Alleviate Hemorrhagic Colitis

Indole is vital for the pathogenesis of EHEC. Hemorrhagic colitis, a bloody type of diarrhea and can lead to a hemolytic uremic syndrome, is caused by the human pathogen *E. coli* O157:H7. During gastrointestinal infection, *E. coli* O157:H7 is revealed to a series of signaling molecules, containing bacterial signaling molecules like indoles, that have been implicated in the control of phenotypes important to EHEC infection, including virulence and infection (114). Indole decreased gene expression in EHEC implicated in surface colonization and pathogenicity, according to a DNA microarray study of surface-associated EHEC (43).

Indole can be oxidized by oxygenase to produce new interspecific biofilm signals, which affecting the same phenotypic (biofilm production) in a wide range of ways. Indole generated by *E. coli* K-12 and other symbiotic bacteria in the intestine might limit the production of *E. coli* K-12 biofilm and diminish the motility of non-pathogenic *E. coli* by changing the expression of *SdiA*, thus influencing EHEC chemotaxis and adhesion (114). Adhesion assays confirmed that indoles reduce the attachment of EHEC to ECs *in vitro* (43).

In conclusion, indoles attenuated EHEC infection and improved hemorrhagic colitis by changing the expression of EHEC toxicity genes, reducing its motility and biofilm formation, and reducing adhesion.

### 5.3 Indoles Remit Colorectal Cancer

According to mounting data, intestinal indoles, particularly Trp metabolites, appear to play an important role in CRC (37). Indoles stimulate AhR, which can work directly on intestinal stem cells to sustain MUC synthesis and improve intestinal barrier function (115). However, blocking the indole-AhR signaling pathway increased TNF-α, IL-1β, and IL-6 mRNA levels considerably in the inflammation-associated colorectal carcinogenesis model (115). Indoles also boost the production of the anti-inflammatory cytokines IL-10 (31) and IL-22 (58). In addition, tryptamine, the indole derivative, inhibits the enzyme indoleamine 2,3-dioxygenase (IDO1), which is involved in tumor immune tolerance (116). These findings imply that indoles generated by gut flora may slow the progression of CRC.

Alterations in microbial Trp metabolism are also characteristic of CRC. CRC patients had a lower indole/Trp ratio and a greater kynurenine (kyn)/Trp ratio than healthy individuals (115). In CRC, the expression of Kyn and IDO1 increase, and the production of indole decreases (117, 118). Increased IDO1 activity and increased Trp depletion cause activated T lymphocytes to enter a cell cycle arrest, leading to apoptotic T cell death and promoting immunosuppression of the tumor microenvironment increases and indole production decreases (119–121). The decline in indole production attenuated the inhibition of colon cancer. Overall, evidence suggests that the altered microbial Trp-indole metabolic pathway plays a role in CRC pathogenesis. As a result, identifying the role of indoles in CRC pathogenesis is critical for developing possible treatment methods.

### 5.4 Indoles Mitigate Diabetes Mellitus

A higher serum concentration of IPA reduces the possibility of developing type 2 diabetes mellitus and enhances insulin secretion and sensitivity. Studies have found that compared with rats fed the control diet, the fasting glucose level of rats fed the IPA diet was significantly reduced (122). According to additional research, indole can modulate the release of glucagon-like peptide-1 (GLP-1) from mouse fibroblasts in the colon (30).

Indole increases GLP-1 release during a short exposure period, reducing its secretion over a longer period. These action results arise because indole can influence the two critical chemical pathways of L cells. To some degree, indole can inhibit volt-gated K^+^ channels, increase action potentials duration induced by L cells, and cause a large increase in Ca2^+^ entry, which boosts GLP-1 secretion quickly. Indole inhibits NADH dehydrogenase, slowing ATP generation and lowering GLP-1 release over time (30).

Exposure to similar indole concentrations in the human colon can regulate incretin secretion by l-secreting cells and modify GLP-1 released by endocrine cells, which is critical for increasing insulin secretion by pancreatic beta cells, lowering appetite, and slowing stomach emptying.Therefore, indole at the intestinal level may affect appetite (123). At the same time, IPA has a potent ability to resist oxidative stress, suggesting that this metabolite may protect beta cells from metabolic and oxidative stress-related damage and amyloid accumulation (124). Therefore, IPA produced by intestinal microbiota metabolism is protective in against type 2 diabetes.

### 5.5 Indoles Abate Central System Inflammation

A neurological condition caused by an autoimmune response is multiple sclerosis (MS). As a central nervous system (CNS) cell, astrocytes are thought to play an important role in MS progression. Indole can be used as a precursor for synthesising AhR agonist Indoxyl sulfate (IS) in the liver and combining it with a type I interferon (IFN1) signal. Indole produced by the breakdown of intestinal flora bind to IFN1 produced in the CNS, which causes CNS inflammation and is activated and suppressed by AhR signaling in astrocytes. Activating AhR signals in astrocytes inhibits CNS inflammation in animal models of MS experimental autoimmune encephalomyelitis (125).

Indole, a gut microbiota metabolite, can reduce inflammation in the central nervous system by modulating AhR (39). Therefore, targeting gut microbiota associated with indole by regulating endogenous gut microbiota may be an alternative strategy for preventing and treating MS and other neurological diseases.

### 5.6 Indoles Lighten Vascular Regulation

IPA could trigger the PXR, a biomass-activated nuclear receptor in various tissues, comprising the vascular endothelium, to regulate endothelial function. IPA regulates agonist-induced endothelium-dependent relaxation in aorta and pulmonary artery catheters through PXR. This regulation is mainly due to the alteration of nitric oxide produced by endothelial nitric oxide synthase, which is repressed by the IPA-mediated activation of PXR (126). However, an antibiotic treatment that destroys the intestinal microbiota and reduces IPA abundance changes the vasodilator effects of IPA by changing the endothelium PXR pathway. The IPA supplement of microbial metabolism could raise systemic IPA levels as well as stimulate PXR expression, thereby reversing the agonist-induced enhancement of endothelium-dependent vasodilator in the aorta and pulmonary arteries caused by antibiotic treatment (127). The interaction between indole and blood vessels may significantly change the antibiotic treatment related to traditional infectious diseases or colon surgery, resulting in disorder in the microbial community.

## 6 Potential Side Effects of Indoles

Although indoles are essential in improving intestinal and even systemic diseases, they still cause negative effects, such as IS, indolyl-β-d-glucosinolate, and IAA (128). IS is co-metabolized by intestinal flora and host. Intestinal flora decomposes Trp in food with an enzyme to generate indoles, which are then carried by the portal vein to the liver and transformed into IS under the action of cytochrome P450 enzyme and sulfotransferase (129). IS is one of the most important nephrotoxic metabolites, and its nephrotoxicity has been widely confirmed in basic and clinical studies (130, 131). In addition, indoles emerge toxicity in the gastrointestinal systems, nervous and cardiovascular. We show the toxic effects of indoles in **Figure 4**.

IS induces IEC damage by up-regulating IRF1 expression, inhibiting dynamin-related protein 1 expression, and interfering with mitochondrial autophagy flux. IS causes oxidative stress in IEC-6 cells by increasing the release of reactive oxygen species in a concentration-dependent way (132). IS also suppresses NRF2 activation, reducing the antioxidant defense cell system and suppressing heme oxygenase-1, NAD(P)H dehydrogenase, and superoxide dismutase expression. In IS-treated IEC-6 cells, connexin 43 is more prevalent in the cytoplasm and nucleus than at the membrane level, resulting in decreased gap junction communication and cell motility. In addition, nucleoconnexin 43 is associated with reduced cell proliferation and significantly promotes intestinal changes associated with chronic kidney disease (CKD) (133). In IEC-6 cells, IS treatment induced significantly increased TNF-α release, cyclooxygenase-2 and inducible nitric oxide synthase production, and nitrotyrosine synthesis, suggesting that IECs are targets of IS-induced intestinal inflammation (134). Colons incubated with IS showed reduced contractility, suggesting that the toxin may have deleterious effects on colonic smooth muscle cells and cause injured intestinal motility (135).

The microbiota metabolite, IS, has a pathogenic role in developing CNS diseases. It is well known that CKD and cardiovascular disease are frequently caused by IS, a protein-bound uremic toxin (136). Additionally, renal insufficiency causes uremic toxins to accumulate in the brain, resulting in aberrant CNS function (137, 138). Secondly, IS can damage neurons and neural stem cells, impair neurotrophic factors and neurotransmitters, and induce oxidative stress and neuroinflammation. For example, by acting on CNS glial cells, IS promotes neuroinflammation and exhibits pro-inflammatory effects (139, 140). Intraperitoneal administration of IS to male C57BL/6 mice undergoing mono-kidney removal (141) revealed an accumulation of IS in blood, prefrontal cortex tissue, and liquor cerebrospinal. In contrast, the mice exhibited behavioral evidence of emotion disorder and neuron degeneration, such as anxiety, depression, and cognitive dysfunction. These Corresponding organic lesions accompanied these behavioral changes. This also suggests a seemingly pathological link between IS and CNS disorders.

In individuals with CKD, IS is linked to cardiovascular and all-cause mortality (142, 143). IS is an essential factor in developing cardiovascular disease in patients with hemodialysis. In hemodialysis, free form IS levels were found that have a positive correlation with Fibroblast growth factor 23 and inversely correlated with C-C motif chemokine 15, complement component C1q receptor, perlecan, bleomycin hydrolase, Cluster of differentiation 166 antigens, and signaling lymphocytic activation molecule family member 5 (144). These proteins serve a vital role in vascular repair and endothelial growth. IS can also cause thrombosis and atherosclerosis by increasing platelet hyperactivity, raising plasma procoagulant levels, and producing procoagulant particles (145, 146). IS affects heart tissue through increased inflammation, cardiac fibrosis, cardiomyocyte proliferation (147).

IS has been linked to the advancement of kidney disease (148), and there is strong evidence that it is harmful when accumulated under renal insufficiency. IS damages proximal tubule cells and induces inflammation and fibrosis development (149–152). Kidney achieves high clearance of IS by renal tubular secretion (153), whereas IS to plasma protein binds over 90%, which is limited by protein binding (154–157), and the plasma level of hemodialysis patients is relatively high, which also suggests the close link with renal disease.

## 7 Discussion and Conclusion

As intestinal bacteria are common metabolites, the important biological role of indoles cannot be ignored. Indoles are directly linked to the homeostasis of intestinal flora and the intestinal tract’s health, so indoles could indirectly affect other systems and the overall health of the human body. A recent study shows that, IAA, an indole derivative, can alleviate ankylosing spondylitis in mice by restoring intestinal microflora balance and decreasing the inflammatory response (158). Indoles have beneficial effects on human health, modulating the intestinal barrier and helping to maintain intestinal homeostasis by activating immune cells to release anti-inflammatory factors such as IL-22 (58), inhibiting the colonization of pathogenic bacteria including EHEC (31), inducing gene expression of TJs and AJ to reduce intestinal permeability (96), and increasing MUC expression to enhance mucus barrier function (22). Mainly, indoles in the regulation of intestinal microecology also play a key role. They inhibit harmful strains of bacteria and alter the virulence of intestinal pathogenic bacteria in a way that affects gene expression, which can help alleviate diseases such as hemorrhagic colitis (114). Simultaneously, indoles and their derivatives are crucial in activating AhR and PXR-mediated anti-inflammatory pathways. Examples include that IPA mediates downregulation of enterocyte TNF-α *via* PXR and upregulates mRNAs encoding for somatostatin to regulate intestinal permeability and intestinal barrier function in inflammation (74). These imply promising therapeutic pathways for indole and its derivatives.

Nevertheless, indole is also a double-edged sword, and a few derivatives also have certain disadvantages. For example, IS generated by the liver metabolism of indole has renal toxicity and cardiovascular toxicity at high concentrations (143), which can cause multiple system dysfunction by promoting oxidative stress, inflammation, and other pathological changes.

There is growing evidence that indoles play a crucial role in intestinal homeostasis and human health, but many questions remain addressed. Many intestinal bacteria were identified to metabolize Trp to indoles. Still, there may be unidentified bacteria, so the use of metabolomics and macro-genomics are needed in the future to further characterize unknown indole-producing bacteria and their associated metabolic pathways. Different indole concentrations exert other physiological functions. In addition, fecal indole concentrations are known in healthy adults, while the concentrations of many indole derivatives in the human intestine and blood remain unknown. Determining the concentrations of indole and its derivatives in different environments is obvious, but a quantitative method specifically for the detection of indole is lacking and simple and rapid assays need to be developed in the future. Most of the studies are based on animal models and involve only one indole. There are limitations in the experimental models and subjects, so further studies on the relationship between indoles and human health are still needed. In the future, there is a need to investigate the effects of multiple indole combinations on host physiology, and there is a need to integrate these findings with the clinical setting to develop new therapies for related diseases.

## Author Contributions

Conceptualization, LL, WL, and XY. Writing – Original Draft Preparation, XY, HL, XZ, and SM. Writing – Review and Editing, XY, HL, XZ, SM, GZ and KA. All authors contributed to the article and approved the submitted version.

## Funding

This work was supported by the National Key Research and Development Program of China (2018YFC2000500); start-up funds from Zhejiang Shuren University (2018R006); National Innovation and Entrepreneurship Program for College Students (202011842025).

## Conflict of Interest

The authors declare that the research was conducted in the absence of any commercial or financial relationships that could be construed as a potential conflict of interest.

## Publisher’s Note

All claims expressed in this article are solely those of the authors and do not necessarily represent those of their affiliated organizations, or those of the publisher, the editors and the reviewers. Any product that may be evaluated in this article, or claim that may be made by its manufacturer, is not guaranteed or endorsed by the publisher.
