# Supplementary material for: Dual Role of Indoles Derived From Intestinal Microbiota on Human Health
Source: Front Immunol. 2022 Jun 17;13:903526. doi: 10.3389/fimmu.2022.903526 (PMC9248744; doi:10.3389/fimmu.2022.903526)
Supplement: Supplementary file 1 [file Table_1.docx]

| Abbreviation contrast table | |
| --- | --- |
| abbreviation | **Complete words** |
| Ahr | aryl hydrocarbon receptor |
| AJ | adherens junction |
| ARE | antioxidant response element |
| ARNT | aromatic hydrocarbon receptor Nuclear Translocator |
| Breg | B regulatory |
| CKD | chronic kidney disease |
| CNS | central nervous system |
| CRC | colorectal cancer |
| *C. sporogene* | *Clostridium sporogenes* |
| DC | dendritic cells |
| DRE | dioxin reaction element |
| DPIELs | double-positive intraepithelial T lymphocytes |
| *E. coli* | *Escherichia coli* |
| EHEC | *enterohemorrhagic* *Escherichia coli* |
| GLP-1 | glucagon-like peptide-1 |
| Hsp90 | heat shock protein 90 |
| IA | indoleacrylic acid |
| IAA | indoleacetic acid |
| IAAld | indole-3-acetaldehyde |
| IAld | indole-3-aldehyde |
| IBD | inflammatory bowel disease |
| IDO1 | indoleamine 2,3-dioxygenase |
| IECs | intestinal epithelial cells |
| IFN1 | type I interferon |
| IFN-γ | interferon-γ |
| IL | interleukin |
| IL-10R1 | IL-10 receptor ligand-binding subunit |
| ILA | Indole-3-lactic acid |
| IPA | Indole-3-propionic acid |
| IPyA | Idole-3-pyruvic acid |
| IS | indoxyl-sulfate |
| kyn | kynurenine |
| *L. acidophilus* | *Lactobacillus acidophilus* |
| LPL | lamina propria lymphocytes |
| MLN | mesenteric lymph nodes |
| MS | multiple sclerosis |
| Muc | mucin |
| NF-κB | Nuclear factor κB |
| Nrf2 | nuclear factor erythroid 2-related factor 2 |
| NSAIDs | non-steroidal anti- inflammatory drugs |
| *P. russellii* | *Peptostreptococcus russellii* |
| PXR | progesterone X receptors |
| STAT | signal transducer and activator of transcription |
| TJs | tight junctions |
| TLR4 | toll-like receptor 4 |
| TNF-α | tumor necrosis factor-α |
| Trp | tryptophan |
| Tr1 | T regulatory cell 1 |
| XAP2 | X-associated protein 2 |
